# Exploring the Potential of Polyvinyl Alcohol–Borax-Based Gels for the Conservation of Historical Silk Fabrics by Comparative Cleaning Tests on Simplified Model Systems

**DOI:** 10.3390/gels12010097

**Published:** 2026-01-22

**Authors:** Ehab Al-Emam, Marta Cremonesi, Natalia Ortega Saez, Hilde Soenen, Koen Janssens, Geert Van der Snickt

**Affiliations:** 1 Department of Conservation, Faculty of Archaeology, Sohag University, Sohag 82524, Egypt; 2 ARCHES Research Group, Faculty of Design Sciences, University of Antwerp, Mutsaardstraat 31, 2000 Antwerp, Belgium; 3 AXIS Research Group, Faculty of Science, University of Antwerp, Groenenborgerlaan 171, 2020 Antwerp, Belgium; 4 Nynas NV, Groenenborgerlaan 171, 2020 Antwerp, Belgium

**Keywords:** gel-cleaning, micro-FTIR spectroscopy, micro-vacuuming, polyvinyl alcohol–borax-based gels, rheology, silk, soot, sponging, textile conservation

## Abstract

Cleaning historical silk textiles is a particularly sensitive operation that requires precise control to prevent mechanical or chemical damage. In this study, we investigate using flexible PVA–borax-based gels to remove soot from silk, i.e., polyvinyl alcohol–borax (PVA-B) gels and polyvinyl alcohol–borax–agarose double network gels (PVA-B/AG DN) loaded with different cleaning agents—namely, 30% ethanol and 1% Ecosurf EH-6—in addition to plain gels loaded with water. These gel formulations were tested on simplified model systems (SMS) and were applied using two methods: placing and tamping. The cleaning results were compared with a traditional contact-cleaning approach; micro-vacuuming followed by sponging. Visual inspection, 3D opto-digital microscopy, colorimetry, and machine-learning-assisted (ML) soot counting were exploited for the assessment of cleaning efficacy. Rheological characterization provided information about the flexibility and handling properties of the different gel formulations. Among the tested systems, the DN gel containing only water, applied by tamping, was easy to handle and demonstrated the highest soot-removal effectiveness without leaving residues, as confirmed by micro-Fourier Transform Infrared (micro-FTIR) analysis. Scanning electron microscope (SEM) micrographs proved the structural integrity of the treated silk fibers. Overall, this work allows us to conclude that PVA–borax-based gels offer an effective, adaptable, and low-risk cleaning strategy for historical silk fabrics.

## 1. Introduction

Silk is an invaluable part of our tangible and intangible cultural heritage, with its earliest use dating back to 5000 years ago in ancient China. Due to its high luster, smoothness, and strength, silk fibers were processed into fabrics, of which a wide range of textile objects were made, including clothing, flags, furniture upholstery, canopies, tapestries, banners, etc. [1,2,3,4,5]. The fibers are produced by the silkworm caterpillar that extrudes twin filaments made of the protein fibroin, bonded with sericin gum (a proteinaceous material) when preparing its cocoon. As this bonding material is responsible for the stiffness of the fibers, sericin is removed in a process called degumming, in which the filaments are separated to obtain two sheen fibers [6,7]. However, in spite of its favorable properties, this natural fiber is vulnerable and known to degrade when exposed to light, heat, microorganisms, air pollutants, high levels of relative humidity, etc., leading to brittleness, loss of mechanical strength, and yellowing [6,8]. For this reason, the conservation and cleaning of silk fabrics is a critical process.

Cleaning textiles, in general, is deemed to be a delicate procedure. Traditionally, conservators can opt between (a) mechanical removal of loose surface soiling, (b) wet cleaning by water, which is usually loaded with surfactants and anti-redeposition agents, commonly referred to as “washing”, and (c) wet cleaning using organic solvents. The latter two methods are commonly employed when removing bonded soiling [7] and involve immersion and rinsing the object [9,10,11]. Due to the irreversibility of the cleaning process, which may cause permanent damage to the object, it is important to assess beforehand the potential impact of this treatment on the fibers and other constituents, such as dyes or metallic threads [12]. Therefore, alternative cleaning methods have been investigated for such special applications: for example, laser cleaning [13,14], hydro-aspiration cleaning [15], and gel cleaning [16].

The main advantage of gels lies in the confinement of the liquids within the structure, minimizing their penetration into the internal structure of the object to be treated. They also limit the lateral diffusion of the liquids on the surface, thus enabling selective and localized cleaning of specific areas [17,18,19]. To date, textile-cleaning research has predominantly focused on the use of rigid gels (specifically agar, agarose, and gellan gum) [16,20,21,22,23,24,25,26,27,28]. A smaller number of studies have also investigated gel-like materials, which are technically considered to be thickeners, such as methyl cellulose, Laponite RD, Xanthan gum, Klucel G, and Carbopol [29,30,31,32]. Nevertheless, both rigid gels and thickeners exhibit several limitations. Regarding rigid gels, it is difficult to achieve a sufficiently close and even contact between the highly textured textile surface and the gel. For this reason, multiple ways were proposed to obviate this issue: for instance, placing weights on the gel (such as glass plates), applying gentle hand pressure, or applying suction through a suction table for a few minutes [16,20,24]. Moreover, after using rigid gels, the formation of tidelines has been reported in some cases. This issue can be resolved by either applying a hydrophobic barrier, such as cyclododecane and decamethylcyclopentasiloxane (D5) [16,31], using a suction table [20], or reapplying the gel on the tidelines [22,28]. The thickeners, on the other hand, have the disadvantage of leaving residues on the treated surface [29,30,31,32].

In light of the aforementioned limitations, this study explores the feasibility of using gel-like materials, specifically polyvinyl alcohol–borax-based (PVA-B) materials, for cleaning textiles. To the authors’ knowledge, these systems have not previously been used in the cleaning of textiles. This class of systems (hereinafter referred to as ‘gels’) are expected to be suitable for this purpose for the following reasons: (a) they are flexible, allowing for better contact with the textured textile surface, (b) they can be applied in different ways, either by simply placing them on the surface or by gently tamping the surface, (c) they have an adjustable and suitable level of liquid retention, and (d) they are proven to leave no residues on the treated surfaces when blended with agarose [19,33,34,35,36]. More details on the cleaning strategy and the selected gels themselves, i.e., polyvinyl alcohol–borax (PVA-B) and polyvinyl alcohol–borax/agarose double network (PVA-B/AG DN), can be found in Section 4.3. For this study, simplified model systems (SMS) also known as mock-ups, were prepared and covered with soot as a contaminant. Soot was selected because it is a common particulate matter that affects cultural heritage objects, including textiles, and its removal is challenging for conservators. The relevance of soot as a significant threat in the context of climate change is, for example, endorsed by the ongoing Horizon 2020 MOXY project that focuses on the removal of this contaminant by means of cold plasma-generated atomic oxygen [37]. Soot results from the incomplete combustion of organic materials, which can be produced in archeological sites, historical buildings, and museums due to numerous activities such as cooking, lighting, or heating, or it can originate from wildfires, building fires, and environmental deposition [24,38,39,40,41,42]. Untreated and undyed silk fabric was selected as the substrate material because it allows for assessment of the material’s response to our cleaning technique(s), offering insights into how these methods may affect the textile’s physical properties, coloration, and structural integrity.

Gel cleaning results were compared to the most prevalent traditional cleaning approach, i.e., micro-vacuuming of the fabrics, followed by sponges, as described in more detail in Section 4.3. Comparisons with aqueous or solvent-based immersion cleaning were not included in this study, as such methods are rarely applied in conservation practice. Silks are typically processed into garments that consist of multiple components, such as linings, interlinings, embroidery, paints, or dyes. Therefore, they can respond unpredictably to full-immersion treatments. These heterogeneous material assemblies make bath cleaning highly risky. Consequently, textile conservators predominantly employ localized cleaning approaches, for which methods such as gel-based systems provide effective and controlled solutions.

The cleaning efficacy was assessed through visual examination, 3D opto-digital microscopy, colorimetric measurements, and machine-learning-assisted (ML) soot counting [43]. The lateral diffusion of the liquids during the application of the gels was traced by loading them with a fluorescent water-soluble dye, “uranine”. This allowed for the evaluation of the tidelines’ formation resulting from the various gel formulations. Rheology measurements were performed on the gel formulations to investigate their viscoelastic behavior and its potential impact on the cleaning results, as well as the handling properties of the gels. Upon the selection of the most suitable treatment, the residues of the cleaning material on the treated SMS were probed by means of micro-FTIR spectroscopy. Moreover, this method also allowed us to assess the chemical stability of the silk fabrics after the treatment. Finally, SEM investigations were carried out to check the structural integrity of the silk fibers.

## 2. Results and Discussion

### 2.1. Visual, Microscopic, and Practical Observations

**Figure 1:**
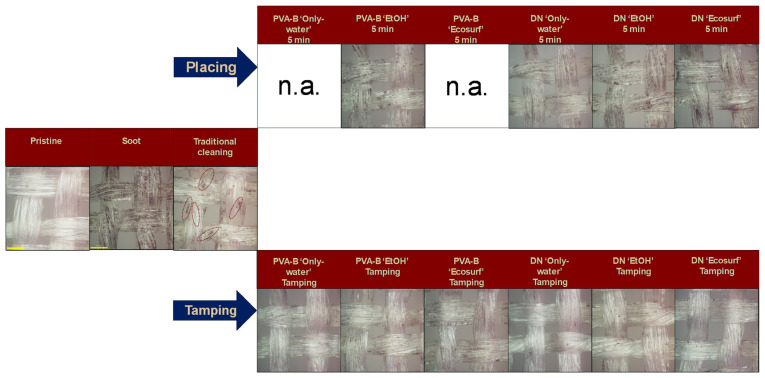
Overview illustrating microscopic images of the SMS treated with the different cleaning approaches adopted in the study: traditional cleaning, placed gels (5 min), and tamping in comparison with the pristine and sooted SMS. No microscopic images were acquired of the gels adhering to the fabric, referred to as (n.a.). The red dash circles indicate the damaged silk fibers in the SMS treated with traditional cleaning. Images corresponding to the SMS treated with the gels placed (1 min) are shown in the Supplementary Materials to improve the overall readability of the figure.

Upon studying the micrographs in Figure 1, it becomes clear that traditional cleaning still leaves a considerable amount of soot not only on, but also in between the fibers. This is due to the mechanical action of the sponge that, next to taking up soot, also moves some of the particles. In this way, the sponge action pushes them deeper into the weave structure, making it harder to clean the remaining soot. This probably explains why, at some point, longer cleaning fails to improve the result. In addition, it was not always possible to achieve a consistent level of cleanliness across all four SMS (each treatment was repeated four times), indicating that it might prove challenging to obtain a homogeneous cleaning result on larger surfaces. More importantly, the micrograph shows mechanical damage, such as the formation of tears and small breakages. As such, these results demonstrate why it is relevant to look for an alternative cleaning approach.

**Figure 2:**
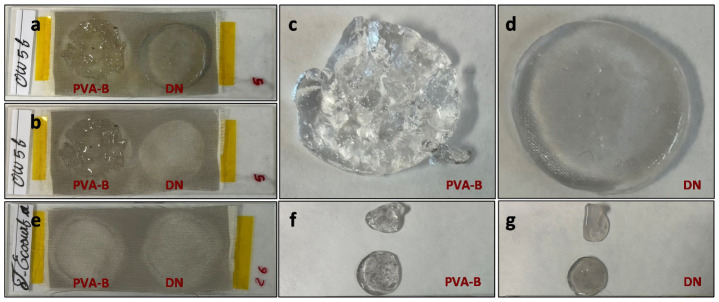
Images illustrating cleaning results (**a**) Sooted silk simplified model systems (SMS) shows a residual layer of polyvinyl alcohol–borax (PVA-B) gel after 5 min application (on the left) and a double network (DN) gel pellet during the application (on the right); both gels were loaded with only water. (**b**) The same SMS after the treatment with PVA-B and DN with only water. (**c**) and (**d**) show the PVA-B and DN gel pellets (ca. 1.5 cm diameter), respectively, after being removed from the same SMS. Note the fabric weave imprint on the DN, signaling the gel’s beneficial flexibility. The PVA-B gel pellet was ruptured upon peeling off, due to the adhesion to the fabric. (**e**) SMS treated by tamping PVA-B and DN gel pellets loaded with Ecosurf. The treated areas are not entirely homogeneous. (**f**) and (**g**) illustrate the PVA-B and DN gel pellets used for tamping the SMS in (**e**), respectively.

The micrographs recorded on the SMS cleaned with gels instantly reveal that the samples cleaned by actively tamping the surface (see bottom row in Figure 1) exhibit less soot than the gels that were placed for one and five minutes (see top row in Figure 1). For the latter placed gels, the five-minute application appeared more effective than the one-minute application. In Figure 1, only the five-minute results are shown; the one-minute results can be consulted in the Supplementary Materials (see Figure S2). Regarding the handling properties of the gels, the PVA-B and DN gel formulations loaded with only water and Ecosurf were easy to handle and to apply on the surface, particularly during the tamping action. Furthermore, they exhibited enough flexibility, allowing for effective contact and adaptation to the fabric weaves when placed, as can be seen in Figure 2d. The gel formulations loaded with EtOH were stiffer and had less surface adaptation. However, for the PVA-B gel formulations with only water and Ecosurf, the gels adhered to the fabric in some cases, leaving a layer of gel residues and causing damage to the fabric. This issue was most pronounced for the longer contact time, but not for the PVA-B formulation with EtOH (see Figure 2a–c). This can be attributed to the insufficient mechanical properties of the PVA-B gels, leading them to rupture during removal from the fabric and consequently leaving behind a residual layer of gel [33,44]. Furthermore, the DN formulations (loaded with only water and Ecosurf) exhibited slightly better shape stability and did not flow onto undesired areas, compared to their corresponding formulations of PVA-B. Based on these results, the areas treated with PVA-B, containing only water and Ecosurf, were excluded from the next evaluation methods in Section 2.2 and Section 2.3. For the other placed gels, the cleaning did not cause any noticeable damage to the fibers but seemed to result in less or equal removal of soot in comparison with traditional cleaning. However, potential differences, also between PVA-B and DN gels, are difficult to assess from the optical images in Figure 1 and Figure S2, and therefore, a more quantitative approach was applied in the following sections.

As mentioned, overall, the tamping gels showed better cleaning performance, whereas adhesion was not observed on any of the tested fabrics. Similarly to placing, none of the tamping treatments caused visible damage to the fibers, except for those that adhered to the fabric. However, the PVA-B and DN loaded with Ecosurf exhibited a less homogeneous cleaning outcome (Figure 2e). Additionally, the PVA-B gel with EtOH was difficult to handle, as it crumbled easily during the tamping action. This aspect renders the gel less suitable for our purposes, not only from a practical/handling point of view, but also because residues can be expected that need additional steps for removal. This issue was less noticeable with the equivalent formulation of the DN, whereas no residues were visually observed in the other areas treated by tamping.

### 2.2. Colorimetric Measurements

**Figure 3:**
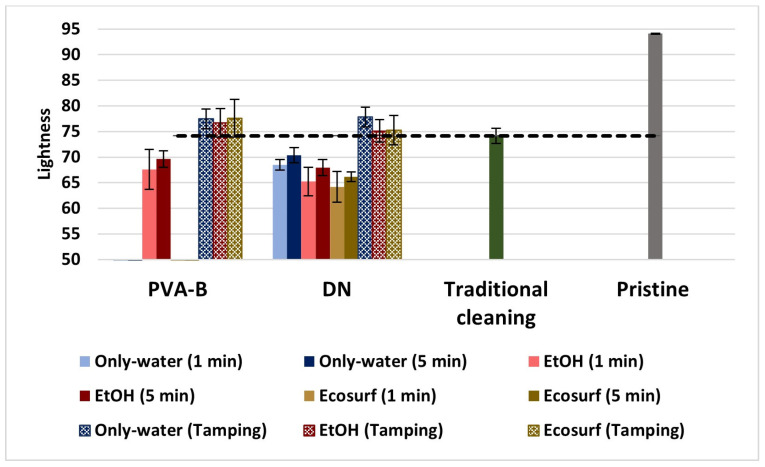
Lightness values recorded on the cleaned SMS, compared with the lightness of a pristine silk fabric. The horizontal dashed line indicates the L value of the traditional cleaning method, serving as a benchmark for evaluating the gel cleaning treatments. The error bars refer to the standard deviations.

Figure 3 shows the lightness (L) values recorded on the samples after gel cleaning and allows for comparison with the (high) lightness of a pristine silk fabric (L = 94.1) and that of a traditionally cleaned SMS (L = 74.1). The graph immediately reveals that not all gel cleaning results in a better outcome than the traditional method. In particular, only the tamping approach yields consistently higher lightness values (L = 75–78), thus indicating a higher degree of soot removal compared with placing (L = 64–70), for both the PVA-B and DN gels. Upon taking a closer look at the tamping methods, it seems that overall, the PVA-B gel formulations exhibited slightly better cleaning results than those of the DN gel formulations.

In contrast, the placed gels clearly underachieve in comparison with the traditional cleaning. Please note that, as mentioned in Section 2.1, the placed PVA-B gel formulations loaded with only water and Ecosurf were excluded, since these leave residues that (a) render the gels unsuitable for cleaning purposes and (b) disturb the colorimetric measurements. The gels that were placed for a longer time for application (i.e., 5 min) provided better cleaning results compared to those that were applied for only one minute. Another noticeable trend is that the soot removal obtained by the gel formulations with only water is slightly better than that of the EtOH-loaded gels, and lastly, the Ecosurf-loaded ones.

In general, the information gained from these measurements substantiates the findings of the visual and microscopical observations, yet they are more detailed.

### 2.3. Machine-Learning-Assisted (ML) Soot Counting

**Figure 4:**
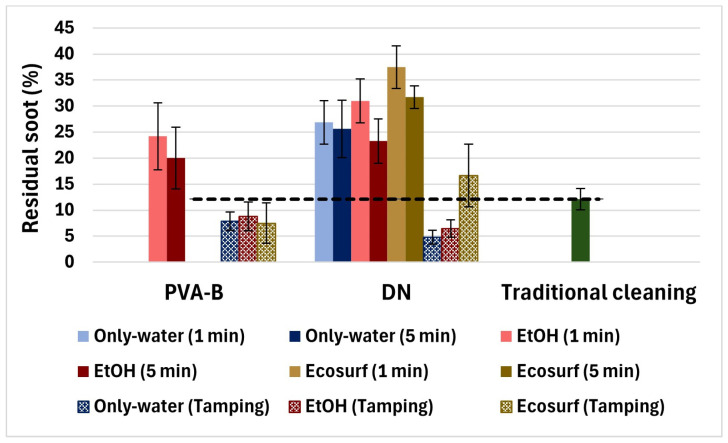
ML soot counting data for all cleaning treatments. The percentage on the *Y*-axis expresses the number of pixels, and thus the amount of surface, that remains covered by soot after cleaning. The dashed horizontal line represents the remaining soot percentage of the traditional cleaning method, providing a benchmark for the gel cleaning treatments. Standard deviations are provided as error bars.

The global trends in the colorimetric results were reflected by the soot counting method, as can be seen in Figure 4. The numbers are, however, considered more accurate and easier to interpret because instead of providing an overall value for lightness, this method isolates individual particles and calculates the actual percentage of the surface that remains covered by soot after cleaning. In this way, it is clear that traditional cleaning performs relatively well, leaving only ca 12% of the surface covered with soot. Again, the tamping approach appears to be more effective than traditional cleaning. In general, tamping with both PVA-B and DN gel formulations leaves a few percent of the soot. An exception to this, conflicting with the earlier colorimetric measurements, was the underperformance of tamping DN doped with Ecosurf, as this removed less soot compared to traditional cleaning. A difference between ML soot counting and colorimetric measurements was anticipated, as previous work showed that the latter is influenced by the gloss of the silk fibers and the texture of the fabric, causing dark shadows [43]. More importantly, the other two DN formulations perform better, with DN ‘only water’ yielding the overall best result when tamping, leaving only ca 5% of the pixels covered with soot.

As expected from Section 2.2, the placing approach, in which the gel formulations were placed for 1 and 5 min, underachieved, showing even lower effectiveness than anticipated, based on the colorimetric measurements. ML soot counting indicated that these methods left almost twice or even three times (for DN with Ecosurf for one minute) as much soot on the surface as traditional cleaning. Within this group, the gels that were placed for five minutes performed better than those that were left for one minute.

### 2.4. Tideline Measurements of Gel Cleaning

**Figure 5:**
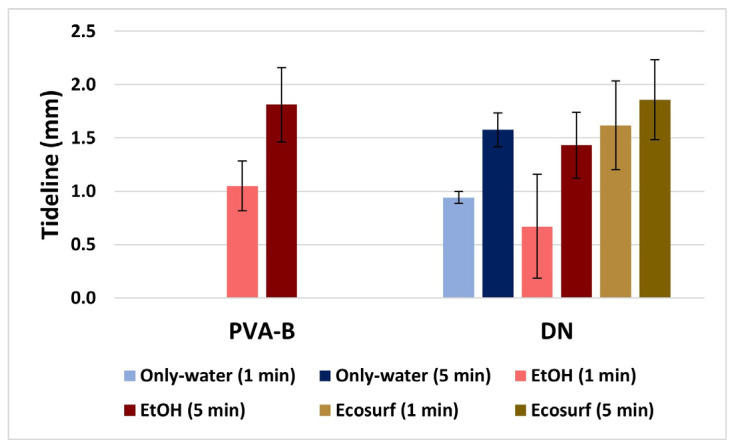
The tidelines (in mm) caused by the different gel formulations when placed on the fabrics for 1 min and 5 min. Error bars indicate the standard deviations.

According to Figure 5, as expected, a longer time for application leads to larger lateral diffusion of liquids on the treated fabrics, meaning larger tidelines. Tidelines did not occur for tamping in view of the short contact time, but they did emerge for all the placed gels, loaded with only water, EtOH, and Ecosurf. For comparing PVA-B with DN, we can only look at the gels loaded with EtOH, as the formulations of PVA-B with only water and Ecosurf were excluded from further testing due to the detrimental potential adhesion to the silk fabric. PVA-B showed larger tidelines than those of the DN. This indicates that the DN has slightly better liquid retention than the PVA-B. When focusing on these DN gel formulations, it can be observed that the gels loaded with EtOH showed reduced tidelines, while those loaded with Ecosurf had the largest. The high evaporation rate of the EtOH probably contributed to reducing the tidelines, whereas the increased tidelines caused by the Ecosurf-loaded gels are related to the fact that this is a surfactant. As such, the application of Ecosurf increases the wettability of the fabric [45], allowing the water to diffuse more on the surface of the treated fabrics.

### 2.5. Rheology Measurements of the Gels

Rheology probes how a material responds to deformation across different time scales. Frequency sweeps in oscillatory mode assess relatively short time ranges, where time corresponds to the inverse of angular frequency. They provide the storage modulus (G′) and loss modulus (G″), describing the elastic and viscous behavior of the material. Gel-like materials typically show a storage modulus that is greater than the loss modulus, indicating a more solid-like response. These measurements help to evaluate a gel’s ability to retain its shape (storage modulus) and its tendency to flow or dissipate energy (loss modulus). Creep tests complement frequency sweeps by probing the long-term deformation under constant stress. In a creep experiment, a constant stress is applied, and the resulting strain is followed over time, revealing the gel’s long-term stability and structural integrity under sustained loads.

**Figure 6:**
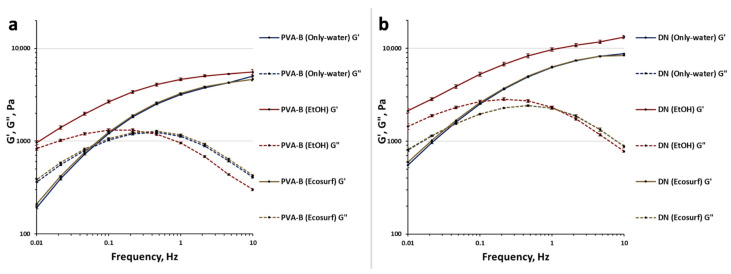
Comparison of both network gels in frequency sweeps. (**a**) PVA-B gels and (**b**) DN gels. The standard deviations are presented as error bars.

**Figure 7:**
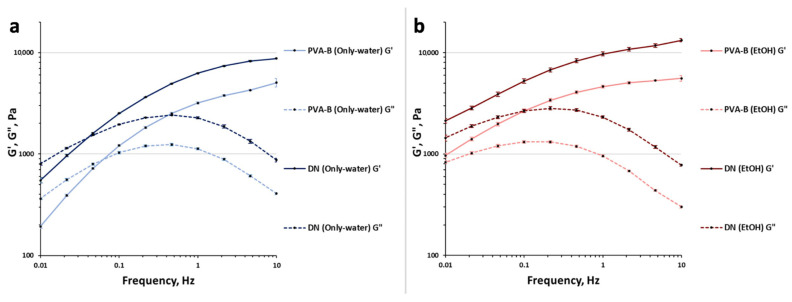
Comparison of frequency sweeps of gels based on PVA-B and DN. (**a**) A diagram shows the gels made with ‘only water’, and (**b**) a diagram shows the gels loaded with ‘30% EtOH’. Standard deviations are represented by the error bars.

In Figure 6, frequency sweeps are illustrated for the PVA-B gels and the DN gels. In both cases, EtOH stiffens the samples, indicating that the EtOH gels deform less when subjected to the same load. The difference in stiffness between both gels increases towards lower frequencies or longer loading times. In addition, for the only water-loaded gels, the storage and loss modulus intersect at a certain angular frequency, which is referred to as the crossover frequency. This frequency gives a characteristic time scale where the material changes from predominantly elastic (solid-like) to predominantly viscous (liquid-like). The higher crossover frequency for the only-water-loaded gels means that these gels start flowing sooner, indicating a quicker-relaxing structure. For the EtOH-loaded gels, the storage modulus is larger than the loss modulus within the applied frequency range, which clearly demonstrates a more solid-like behavior when applying these gels. This explains the noticeable lack of adaptation of the EtOH-loaded gels to the fabric weaves during the placing approach. The direct comparison is illustrated in Figure 7. Ecosurf does not affect the frequency sweeps for both types of gels. However, as was already discussed in the Supplementary Materials, the use of the surfactant had a large effect on the wall slip (as shown in Figure S3).

In addition, all the gels based on the DN gel have a higher stiffness and lower crossover frequency compared to the corresponding PVA-B gels (see Figure 6). This stiffness provided the rigidity of the DN gels, allowing good handling during their application. On the other hand, they were still characterized by enough flexibility for adaptation to the surfaces. Additionally, the balance between the stiffness and flexibility of these DN gel formulations facilitated their handling in the tamping action. This was pronounced in the DN gels with only water and Ecosurf.

**Figure 8:**
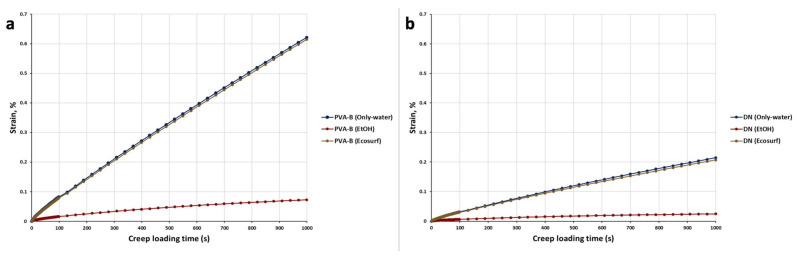
Creep curves of both (**a**) PVA-B and (**b**) DN gel formulations.

As we are exploring the cleaning efficacy of the gels over different application times, the gels were tested within a 15 min time frame to evaluate their shape stability during relatively long times when applied on the fabric. According to Figure 8, the creep tests showed similar trends, as was already obvious from the frequency sweeps. The creep curves of the PVA-B networks are steeper, relating to more deformation within 15 min and a lower viscosity. Again, for both network types, the EtOH increases the viscosity, which significantly minimizes the adaptation of the gel. On the other hand, the Ecosurf has no measurable effect on the creep curves, as illustrated in Figure 8. Overall, the DN formulations exhibit improved shape stability over the 15 min time frame compared to the PVA-B gels, resulting in good shape retention on the desired area and only limited lateral flow. Nevertheless, especially for the only water and Ecosurf gels, the observed deformation enables an intimate contact with the fabric, which is necessary for effective cleaning.

### 2.6. Selection of the Most Suitable Cleaning Treatment

Traditional cleaning serves as our benchmark for comparison, even though it is unsuitable because it causes serious damage to the fabrics. We aim for cleaning treatments with better (a) cleaning efficacy, (b) ease of handling, and (c) safety, with non-damaging performance on the silk. The incremental evaluation methods adopted in this study allowed for discrimination between the different cleaning treatments. In this regard, the treatments involving the placement of PVA-B containing only water and Ecosurf were omitted due to the detrimental adhesion to the surface, which clearly left gel residues on the fabric. Based on the results of the colorimetric measurements and the ML soot counting method, it was clear that the tamping approach provided better cleaning than that of the placed gels. Given also that the placed gels formed tidelines, this made them less suitable than tamping with the gels. Among the gels applied by tamping, the gels loaded with Ecosurf provided less homogeneous cleaning results. According to the ML soot counting, the gels containing only water and EtOH showed good soot removal: specifically those associated with the DN gel. Since PVA-B gels are known to exhibit inferior mechanical properties compared to the DN [33,44], this renders the DN loaded with only water and EtOH as the treatment with the best cleaning efficacy. However, the gels loaded with EtOH had lower handling properties, as they are too stiff and they crumble, making the tamping action difficult. Consequently, the DN gel loaded with only water was considered the most suitable cleaning treatment. A summary of the selection criteria can be found in Table 1.

### 2.7. Micro-FTIR Detection of Gel Residues on the Selected Treatment

**Figure 9:**
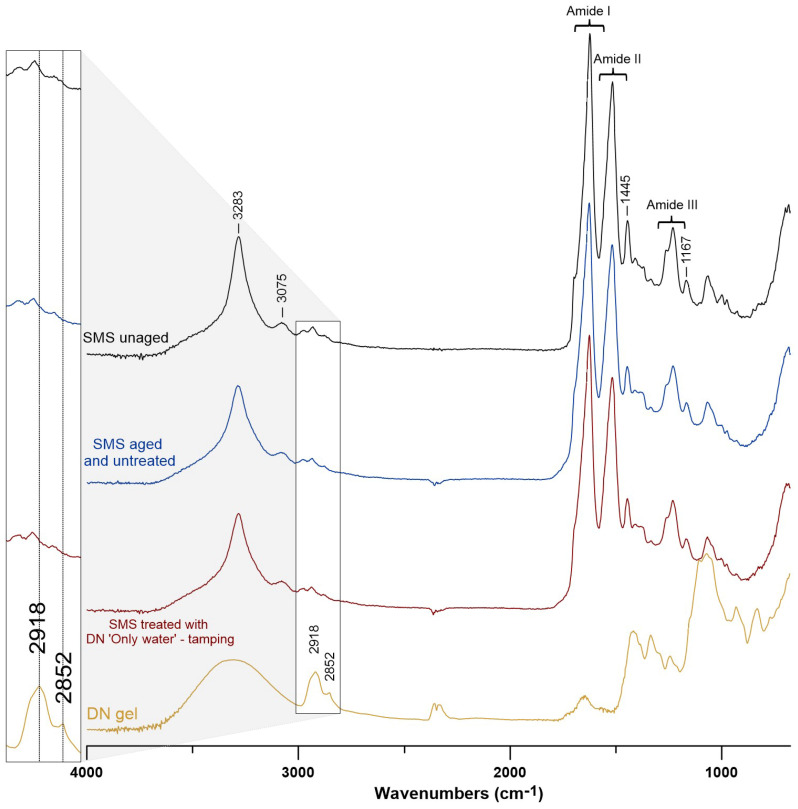
ATR-FTIR spectra of unaged SMS, aged-untreated SMS, SMS tamped with DN loaded with only water, and DN gel. A detail of the spectra in the 2800–3000 cm^−1^ range, shown on the left, indicates that the CH_2_ vibrations of the DN gel (at 2918 and 2852 cm^−1^) are absent in the spectrum of the treated SMS.

Since the tamping treatment using the DN gel loaded with only water provided the best soot removal, micro-FTIR spectroscopy was used to measure the treated surface for gel residues. First, the ATR-FTIR spectra of the untreated SMS silk show the characteristic peaks of silk, as illustrated in Figure 9. The N–H stretching vibrations of the amide A and amide B appear at ~3283 and 3075 cm^−1^, respectively. The main band corresponds to amide I in the 1700–1590 cm^−1^ region, which is attributed to C=O stretching vibrations. Amide II in the 1590–1460 cm^−1^ region corresponds to N–H bending and C–N bending vibrations, while amide III at 1280–1190 cm^−1^ represents the N–H bending and C–N stretching vibrations [46,47,48]. Additionally, the peaks at ~1445 and 1167 cm^−1^ are attributed to the vibrations of CH_2_ and CH_3_ bending in alanine, and C–N stretching in tyrosine, respectively [49]. It is worth noting that the collected spectra also show indications of degradation due to artificial aging. One of the major changes can be detected in the region of 1775–1700 cm^−1^ due to the formation of free carbonyl moieties [2,47,49,50], as shown in Figure 9 and Figure S4. Other alterations in the amide groups can be observed, such as differences in the relative intensities of the amide I band: in particular, those at 1698 and 1653 cm^−1^. In addition, the maximum of the amide I shifted to a higher wavenumber, at 1626 cm^−1^, along with changes in the relative intensities of the amide III bands [47,49]. These measurements were performed to document the chemical changes that were already present before the treatment.

Regarding the gel residues, we decided to focus on the 2800–3000 cm^−1^ range because most of the ATR-FTIR peaks of the DN gel overlap with those of the silk, particularly at lower wavenumbers (see Figure 9). In this range, the DN exhibits the characteristic CH_2_ asymmetric and symmetric stretching vibrations at 2918 and 2852 cm^−1^ [51,52]. The spectra collected from areas treated with the DN gel ‘only water–tamping approach’ showed a clear absence of the peaks of the gel. Additionally, these spectra are similar to those of the untreated silk, indicating that the gel caused no detectable chemical changes in the silk. While these measurements focused on the selected treatments, further insights into the detection of PVA-B gel and Ecosurf residues on the treated SMS are provided in the Supplementary Materials.

### 2.8. SEM Observations on the Selected Treatment

**Figure 10:**
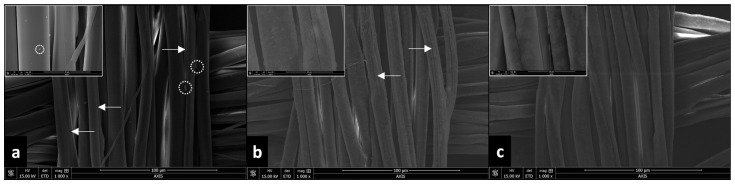
SEM micrographs of different silk SMS; magnified details of the micrographs are shown in the top left corner of each image. (**a**) Artificially aged silk showing longitudinal cracks, ‘arrows’, and sub-micrometric debris, ‘circles’. The magnified image exhibits details of the debris and the cracked fibers. (**b**) Untreated, artificially aged silk covered with highly web-like distributed soot particles. The arrows refer to the longitudinal cracks. More details of the soot particles can be seen in the magnified micrograph. (**c**) Silk treated by tamping with DN ‘only water’; fewer soot particles can be observed compared to (**b**).

The morphology of artificially aged silk (free of soot) was documented to provide a reference for pristine conditions. The aged silk fibers exhibited typical characteristic degradation features that were noticeable through electron scanning microscopy, such as sub-micrometric debris on the surface and longitudinal cracks, which are irregularly distributed across the samples in Figure 10a,b [47,53]. In the soot-contaminated SMS, the deposits displayed a homogeneous, web-like distribution covering the fibers, as can be seen in Figure 10b. Examination of the silk cleaned with the selected DN ‘only water’ gel applied by tamping (Figure 10c) showed no morphological or structural alterations that were attributable to the cleaning procedure, as indicated by the preserved integrity of the fiber surface. Furthermore, the treatment gently and selectively removed a considerable amount of soot particles. A thin, discontinuous layer of residual soot contaminant remained in some areas. Nevertheless, this can be considered acceptable, given the fragility of the substrate.

## 3. Conclusions

In this research, we explored the PVA-B-based gels as a potential cleaning tool for removing soot from aged silk fabrics and compared their performance with that of the most prevalent traditional cleaning approach, i.e., micro-vacuuming combined with sponge rubbing. Specifically, PVA-B and PVA-B/AG DN gels were tested, either by placing or tamping the sooted SMS. The comprehensive evaluation underscored that actively *tamping* the surface with a gel results in a higher soot removal than placing the gel and leaving this passively in contact with the surface for several minutes. However, it should be noted that using suction tables might enhance the cleaning efficacy of the placed gels and reduce/avoid the formation of tidelines, but this was not further explored in this study.

Tamping with a DN gel only loaded with water proved to be the most effective method, leaving only ca 5% of the surface SMS covered by soot, whereas traditional cleaning only reached ca 12%. In addition, this gel formula showed good flexibility, permitting better handling during the tamping action. The outcome was obtained by combining visual and microscopic inspection, colorimetric measurements, ML soot counting, and rheology investigations. Micro-FTIR analyses confirmed the safety of the selected gel treatment, providing a gel-residue-free surface with no impact on the fiber. Additionally, SEM examination revealed no detectable damage to the fibers. This study opens the door to employing PVA-B-based gels as a promising cleaning tool for historical textiles, given their flexibility, versatility, and safety compared to traditional contact-cleaning methods.

It should be noted that this study was limited to a relatively small corpus of SMS, although reproducibility was optimized by repeating each cleaning method on four separate samples, collecting data from five areas, and calculating average values. In addition, the tests were performed on undyed silk, implying that any potential effects of the sensitive dyes were not taken into account. Last but not least, tests were performed on artificially aged silk, which is assumed to be different from historical silk. However, although further investigations would be beneficial, this theoretical assessment on simplified model systems does prove that the method of tamping PVA-B/AG DN gels loaded with only water is a valuable addition to the textile conservator’s toolbox when the cleaning of historical silk fabrics is considered.

## 4. Materials and Methods

### 4.1. Materials

Silk was acquired from IDEEN.com (08 Pongee silk, 100% silk, natural white, 140 cm width, 50 g/m^2^, prewashed, unsized, and untreated). Polyvinyl alcohol (*PVA*) (98.0–98.8% hydrolyzed, M.W. 146,000–186,000) was supplied by Acros Organics (Geel, Belgium). Di-Sodium tetraborate decahydrate (*borax*) (ACS, ISO reagent) was purchased from Merck (Overijse, Belgium). Agarose LE (*AG*) was acquired by Gentaur Europe BVBA (Kampenhou, Belgium). Ethanol (*EtOH*), eurodenatured 99%, was obtained from VWR (Leuven, Belgium). Ecosurf EH-6 (*Ecosurf*), uranine, and shellsol D40 were supplied by Kremer Pigmente (Aichstetten, Germany).

### 4.2. Preparation of Simplified Model Systems (SMS) and Artificial Aging

The SMS were prepared by cutting swatches of Pongee silk. Each swatch measured 2.5 × 5 cm, with a consistent warp and weft orientation. Before cutting the swatches, the textile was prewashed in deionized water at 40 °C. Soot from a direct combustion source was used as the contaminant, following the smoke-drum method developed in [37]. This approach was specifically designed to ensure controllable, reproducible, and uniform soot deposition. In brief, the soot deposition process was performed in a soot drum, involving the burning of a dense cotton wick soaked in Shellsol D40. The rising smoke deposited soot onto a circular array of SMS mounted on metal slides. To achieve a uniform soot distribution, the SMS were rotated horizontally at 17 rpm using an electric motor. The entire deposition setup was enclosed in a metal cylinder to minimize the flame flickering caused by air currents. After 40 min of deposition, the SMS were removed from the slides and transferred to glass microscope slides to facilitate handling (see Figure S1). Next, the SMS were subjected to artificial aging in the weathering chamber Xenotest 440 from Atlas Material Testing Technology GmbH (Linsengericht, Germany). The aging parameters were determined based on preliminary tests, which were adapted from [54]. The aging process lasted for 10 days, with the chamber maintained at a temperature of 50° C, while the black standard temperature (BST) reached up to 105.5 °C. The relative humidity (RH) was set at 80%, and the irradiance was controlled at 80 W/m^2^ within the 300–400 nm wavelength range.

### 4.3. Gel Preparation and Cleaning Strategy

**Scheme 1:**
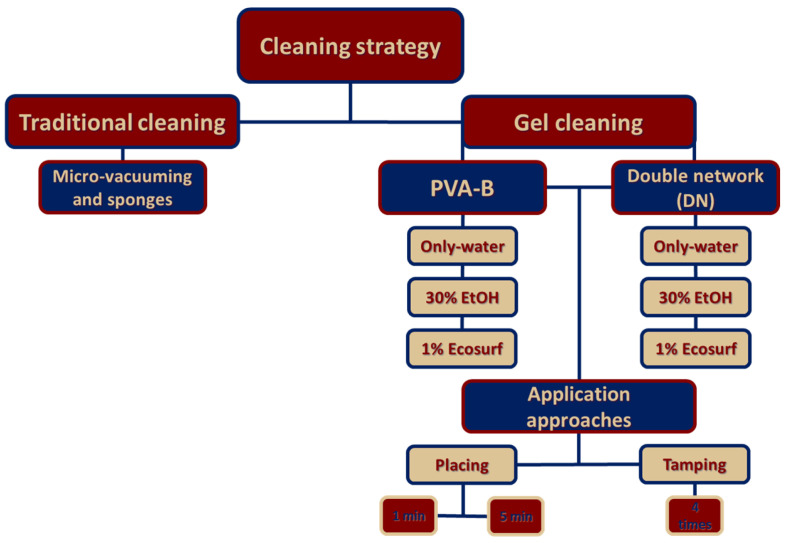
The elements of the cleaning strategy.

Two types of gels were selected for the experiment: namely, PVA-B and PVA-B/AG double network (DN) [33,35]. As demonstrated in Table 2, three variations were prepared: next to the non-loaded gels (hereinafter referred to as ‘only water’), a part of the gels was loaded with either a solvent (i.e., 30% EtOH) or a surfactant (i.e., 1% Ecosurf). Both modifications have been proposed in the surface cleaning literature, with EtOH being a widely used solvent to remove soiling, including soot [36,39], and Ecosurf being a biodegradable and effective wetting non-ionic surfactant [55,56,57]. In this way, the study included six different gel formulations in the evaluation process, as illustrated by Scheme 1. The preparation of the gels was executed as described elsewhere [33]. For the tideline experiment, a separate set of fluorescent gels was prepared, with the abovementioned formulations but adding 0.005% of uranine to the PVA stock solution.

It is worth mentioning that this cleaning strategy was designed based on preliminary trials conducted on silk SMS to assess the feasibility of the treatments and to optimize the various parameters. The six gel formulations were applied on the sooted silk SMS with two different approaches. The first approach involved simply *placing* a circular pellet of gel, ca. 1.5 cm in diameter and ca. 0.3 cm in thickness, on the SMS, as illustrated by Figure 2a. Prior to application, each gel pellet was first placed on a flat surface for ca. 5 min and pressed gently several times to remove any air bubbles from the surface of the gel; the latter may cause inhomogeneous cleaning. The gel pellets were also gently pressed on the surface of the SMS to ensure good contact between the gel and the fabric. Two different contact times, i.e., one and five minutes, were employed for each of the six gel formulations in order to evaluate the effect of short and relatively long application times on the cleaning treatments and the formation of tidelines. Preliminary trials indicated that a longer contact time (10 min) (a) did not improve cleaning but instead resulted in (b) adhesion between the gel and the fabric, leading to residues, and (c) larger tidelines. In view of these relatively long contact times, fluorescent uranine was included in a different set of these gels for tracking potential tideline formation. The second approach involved gently *tamping* the gel pellet on the SMS. To avoid redeposition of the soot that was picked up by the gel, a fresh side of the pellet was used after each tamp. Four times tamping, using two gel pellets, appeared to be enough to remove the soot (see Figure 2e–g). Each time tamping, the gel pellet was in contact with the surface of the fabric for ca. one second. As the very short contact times allowed for avoiding tideline formation, these gels, applied by tamping, were not loaded with fluorescent uranine.

The outcome of the above cleaning strategy was compared with the results of a well-established cleaning protocol for soot removal from fire-damaged silk, in accordance with the published studies [38,39,41]. The cleaning procedure was carried out in two sequential steps. First, loose soot was removed by micro-vacuuming with a museum vacuum cleaner (Romex, HEPA, BlowVac Special Electronic 555-EDS-S-E GS), operated 2–5 mm above the surface and assisted by a soft-bristle brush. Second, surface cleaning was performed by hand-rubbing a latex-free, high-density polyurethane foam sponge (Deffner and Johann^®^). Minimal pressure was applied with each stroke, as the aged silk was highly sensitive to repeated mechanical action. On average, cleaning a 2.5 × 5 cm area required approximately 10–15 min. The two steps were performed until no additional soot removal was noticed by the naked eye. It is also worth noting that four SMSs were cleaned using each cleaning treatment (i.e., traditional cleaning and gel cleaning), to enhance the reproducibility of the tests.

### 4.4. Evaluation of the Cleaning Treatments

The treatments were evaluated iteratively, with each stage employing a more refined and precise method. The evaluation followed a funnel approach in which less effective/damaging cleaning treatments were excluded from the following stage of the process. Initially, visual and stereomicroscopic-aided inspection was used to evaluate the cleaning efficacy, the ease of application, and the potential presence of noticeable residues of the cleaning material or damage to the substrate material. The remaining methods were evaluated in a second round involving classic colorimetric measurements, followed by a machine-learning-assisted soot counting on micrographs to evaluate the soot removal of the treatments. Since the placed gels formed tidelines, it was decided to determine the width of these tidelines on the treated silk fabrics as a figure of merit. The cleaning treatment that was finally selected was measured using micro-FTIR to detect potential gel residues and to investigate chemical changes to the silk that may have occurred after the treatment. Finally, high-magnification SEM inspections were performed to investigate the fiber integrity after the treatment.

### 4.5. Colorimetric Measurements

The colorimetric measurements were performed with an AvaLight-DH-S-BAL deuterium halogen light source (215–2500 nm) and an AvaSpec-2048L spectrophotometer from Avantes (Apeldoorn, The Netherlands). CIELab values were collected using the standard illuminant D65 and the 2° standard observer. All the measurements were processed by Avasoft 8 software. To collect the CIELab data, each SMS was subjected to five measurements on the cleaned area, with a total of 4 SMS for each cleaning treatment. All the measurements were averaged, and the standard deviations were calculated. Since the L* coordinate represents the lightness of the measured area (black = 0 and white = 100), this was considered to be an appropriate value to evaluate the removal of the black soot from (off-)white silk [40]. The acquired data from the cleaning treatments were compared with those of the pristine SMS. Better soot removal was achieved with higher L values. It is worth noting that no significant changes were detected in the b* coordinate, proving that there was no noticeable yellowing after the different treatments. Therefore, the L* coordinate alone was taken into account in this study.

### 4.6. Optical Microscopy and Machine-Learning-Assisted Soot Counting

**Figure 11:**
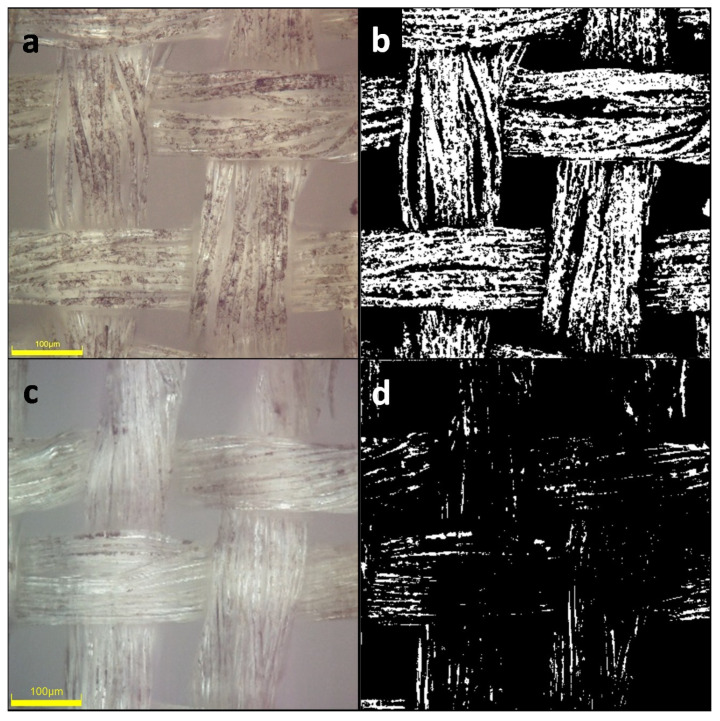
Two examples of image segmentation. (**a**) A micrograph recorded on a sooted SMS before cleaning. (**b**) The corresponding segmented image of the same area, where white pixels represent the remaining soot particles (soot count = 39.4%). (**c**) A micrograph recorded on an SMS after cleaning by tamping the surface with a DN gel, loaded with only water. (**d**) The segmented image of the same area (soot count = 8.4%).

ML Soot counting was executed on the images recorded with a 3D opto-digital stereo microscope (Olympus DSX510, Hamburg, Germany) equipped with Extended Focal Imaging (EFI), at 40× magnification, with an image size of 4800 × 3600 pixels. As illustrated by Figure 11, the images always included the intersection of two weft and two warp threads. In an attempt to enhance quantitative cleaning evaluation, an ML-based image segmentation method was applied to this set of images to identify and count the remaining soot particles after cleaning, according to a recent proposed methodology [43]. In particular, the micrographs were processed in Fiji—an image processing package (Fiji version 2.17.0, ImageJ v1.54p, build date 17 February 2025)—using the WEKA Trainable Segmentation (TWS) plug-in (http://imagej.net/Trainable_Weka_Segmentation, accessed on 17 December 2025). The resulting segmented images were subjected to automatic particle counting, which yielded the relative percentage of pixels corresponding to soot, as shown in Figure 11. This parameter was used to quantitatively assess the performance of the different cleaning treatments, with higher percentages of residual soot indicating less effective cleaning. For each cleaning treatment, four SMSs were cleaned, and five micrographs were acquired for each of them. In this way, for each cleaning treatment, the outcome was based on an average of 20 values.

### 4.7. Tideline Measurements of Gel Cleaning

Technical photography of the set of SMS treated with the six gel formulations (see Table 2) was carried out with a digital camera (Canon 5D Mark II, Amstelveen, The Netherlands) under UV light, using Broncolor Flash Tube 3200 J lamps for Unilite (Allschwil, Switzerland). The wavelength of the UV light was set to 365 nm by using a filter cap on the light source. As mentioned, the gels were loaded with uranine as a fluorescent tracer to monitor liquid penetration laterally into the fibers. The tideline marks were subsequently measured with ImageJ (version 1.54g) by setting the scale and recording the tideline width at five points for each cleaned area. Thus, for each cleaning treatment, the average width of a total of 15 measurements was calculated, as well as its standard deviation.

### 4.8. Rheology Measurements of the Gels

Rheology characterizes the viscoelastic behavior of the gels, describing how their stiffness, elasticity, and flow properties change with frequency or loading time. Because flexibility is a key feature that makes PVA-B-based gels suitable for textile treatment, rheological analysis can help to clarify the handling properties of the six formulations and their ability to conform to the fabric topography. The rheological data were recorded with a controlled stress rheometer (Anton Paar MCR 500, Graz, Austria), using a 25 mm plate–plate geometry. The standard smooth plates and specially manufactured upper and lower serrated plates were used. The serration consisted of a pyramidal structure, with individual pyramids measuring 0.5 mm in length and width and 0.1 mm in height. For the smooth plates, tests were recorded with a gap of 1 mm. For the serrated plates, it was set at 2 mm to reduce possible errors introduced by the serration. All tests were conducted at 25 °C. When loading the samples in the rheometer, they were trimmed 50 microns above the desired gap setting, and afterwards, the gap was decreased to its measuring position. The following measurements were recorded:

- Stress sweeps to determine the linear viscoelastic region and the strength of the crosslinks.
- Frequency sweeps, covering a range from 10 Hz to 0.01 Hz, at strain levels well within the linear viscoelastic range.
- Creep measurements, covering a time range of 15 min at a fixed stress level of 8 Pa to evaluate the behavior at longer loading times.

Each test was recorded at least in duplicate, using different gel specimens. For some of the gels, indications of wall slip were noted with the standard smooth plates. Wall slip is an artifact in rheology that occurs when the material slides along the surface of the rheometer tool instead of deforming uniformly. It occurs when a thin, weak, or even liquid layer forms against the plate surface that differs from the bulk sample. These gels displayed an unexpectedly high stress sensitivity and, consequently, a very limited linear viscoelastic strain region. Additionally, the affected samples did not yield good repeatability levels in any of the tests. Some other gels, on the contrary, showed the expected rheological behavior and good repeatability levels; these gels did not display wall slip. For this reason, all further tests discussed in this study were recorded using the serrated plates. More information about the issue with the smooth plates is explained in the Supplementary Materials.

### 4.9. Micro-Fourier Transform Infrared (Micro-FTIR) Spectroscopy

A Nicolet™ iN10 MX Infrared Imaging Microscope (Thermo Scientific™, Zaventem, Belgium) was used to detect the presence of gel residues that could be formed by the selected cleaning treatment. The microscope was equipped with a mercury cadmium telluride (MCT) nitrogen-cooled detector, and the measurements were executed using Omnic Picta 1.9 software. The spectra were collected in ATR mode, using a germanium (Ge) crystal in the 4000–675 cm^−1^ region, where 128 scans were recorded at a resolution of 4 cm^−1^. Line scans were performed on the SMS—unaged silk, aged silk, and gel-treated silk—using a spot size of 30 μm^2^ and a step size of 30 μm^2^, resulting in a total of 60 points for each SMS. To ensure reproducibility, the presented data are based on an average of at least 30 spectra for each SMS. All the spectra are presented in their raw form, without any manipulation such as smoothing, baseline correction, or other post-processing treatments.

### 4.10. Scanning Electron Microscopy (SEM)

For the SEM investigations, the silk SMS were first gold-coated using the low-vacuum sputter coater (Leica EM ACE200, Leica Microsystems GmbH, Wetzlar, Germany), with a thickness of 4.7 nm. Later, they were examined under a field emission gun–environmental scanning electron microscope (FEG–ESEM) (FEI Quanta 250, Hillsboro, OR, USA). The SEM images were collected using secondary electrons mode (SE) with a working distance of 10 mm, an accelerated voltage of 15 kV, and a sample pressure of 10^−4^ Pa.

## Figures and Tables

**Table 1:** Summary of the evaluation of the various cleaning treatments.

Cleaning Treatment Methods			Soot Removal	Unsolicited Effects	
Traditional cleaning			+/−	Damages the fabric	
Gel cleaning					
Application approach	Gel type	Cleaning agents			
Placing	PVA-B	*Only water*	−	Adheres to the fabric	Gels residues
		*EtOH*	−	Forms tidelines, stiff, and has less surface adaptation	
		*Ecosurf*	−	Adheres to the fabric	Gels residues
	DN	*Only water*	−	Forms tidelines	
		*EtOH*	−	Forms tidelines, stiff, and has less surface adaptation	
		*Ecosurf*	−	Forms tidelines	
Tamping	PVA-B	*Only water*	+	Less mechanical strength compared to its corresponding formulation with DN	
		*EtOH*	+	Stiff, crumbles, and is hard to handle	
		*Ecosurf*	+	Inhomogeneous cleaning	
	DN	*Only water*	++	None	
		*EtOH*	+	Stiff, crumbles, and is hard to handle	
		*Ecosurf*	−	Inhomogeneous cleaning	

Green = very good cleaning treatment, light green = good cleaning treatment, yellow = fair cleaning treatment, and red = poor cleaning treatment.

**Table 2:** Component concentrations for the six gel formulations.

	PVA	Borax	AG	EtOH	Ecosurf	Water	Visual Appearance of the Gels
PVA-B ‘Only water’	4%	1%	-	-	-	95%	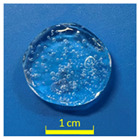
PVA-B ‘EtOH’	4%	1%	-	30%	-	65%	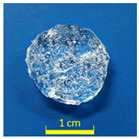
PVA-B ‘Ecosurf’	4%	1%	-	-	1%	94%	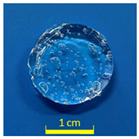
DN ‘Only water’	4%	1%	1%	-	-	94%	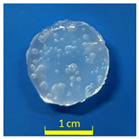
DN ‘EtOH’	4%	1%	1%	30%	-	64%	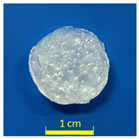
DN ‘Ecosurf’	4%	1%	1%	-	1%	93%	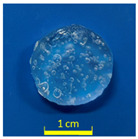

## Data Availability

Data are contained within the article and the Supplementary Materials.

## Supplementary Materials

The following supporting information can be downloaded at: https://www.mdpi.com/article/10.3390/gels12010097/s1, Figure S1: (Top) Aged, not-sooted silk fabric mounted on a glass microscope slide, ‘pristine’. (Bottom) Aged, sooted silk fabric mounted on a glass microscope slide; Figure S2: Overview illustrating microscopic images of the SMS treated with the gels placed for 1 min compared to pristine, soot-covered, and traditionally cleaned SMS. No microscopic images were acquired of the gels adhering to the fabric; Figure S3: (a) and (b): Comparison of smooth and serrated plates in frequency for two hydrogel formulations. (c) and (d): Comparison of stress sweeps for the same hydrogel formulations; Figure S4: ATR-FTIR spectra of artificially aged (blue) and unaged silk (red) in the range of 1800–1100 cm^−1^; Figure S5: ATR-FTIR spectra of untreated SMS, SMS tamped with PVA-B loaded with only water, and PVA-B gel. A detail of the spectra in the 2800–3000 cm^−1^ range is shown on the left. It indicates that the characteristic peaks at 2910 and 2861 cm^−1^ of the PVA-B gel are absent in the spectrum of the treated SMS, as is the intense peak at 1107 cm^−1^ indicated by the dashed line; Figure S6: ATR-FTIR spectra of untreated SMS, SMS treated with DN with Ecosurf, and Ecosurf EH-6. The treated SMS does not show the definitive peak of the Ecosurf at 1098 cm^−1^.

## Abbreviations

The following abbreviations are used in this manuscript:

Ecosurf	Ecosurf EH-6
EtOH	Ethanol
ML	Machine-learning-assisted
Micro-FTIR	Micro-Fourier Transform Infrared
PVA-B	Polyvinyl alcohol–borax
PVA-B/AG DN	Polyvinyl alcohol–borax/agarose double network
SEM	Scanning electron microscope
SMS	Simplified model systems
