# Supplementary material for: Exploring the Potential of Polyvinyl Alcohol–Borax-Based Gels for the Conservation of Historical Silk Fabrics by Comparative Cleaning Tests on Simplified Model Systems
Source: Gels. 2026 Jan 22;12(1):97. doi: 10.3390/gels12010097 (PMC12840747; doi:10.3390/gels12010097)
Supplement: Supplementary file 1 [file gels-12-00097-s001.zip › gels-4079237-supplementary.pdf]

gels

MDPI

Supplementary materials

Exploring the Potential of Polyvinyl Alcohol–Borax-Based Gels for the Conservation of Historical Silk Fabrics by Comparative Cleaning Tests on Simplified Model Systems

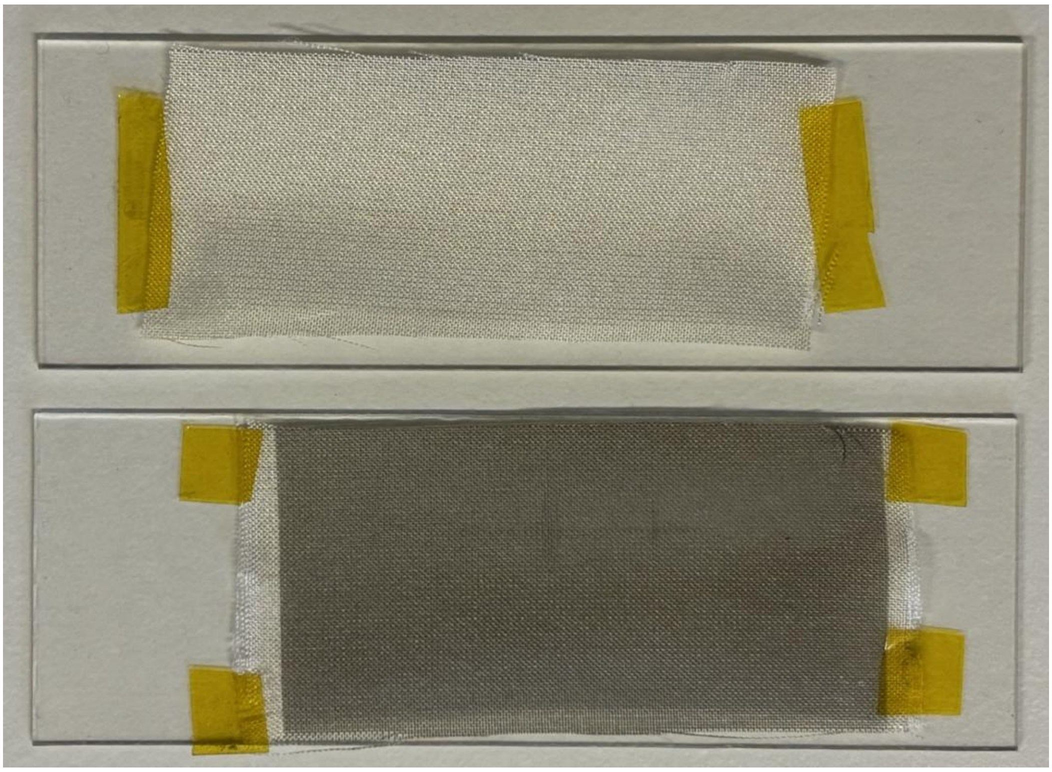

Figure S1: (Top) Aged, not-sooted silk fabric mounted on a glass microscope slide, 'pristine'. (Bottom) Aged, sooted silk fabric mounted on a glass microscope slide.

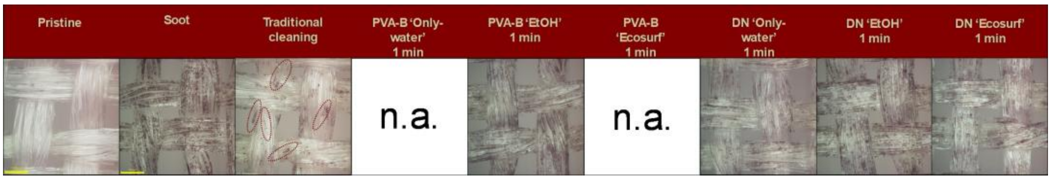

Figure S2: Overview illustrating microscopic images of the SMS treated with the gels placed for 1 min compared to pristine, soot-covered, and traditionally cleaned SMS. No microscopic images were acquired of the gels adhering to the fabric.

Rheology measurements (optimization of the plates)

A comparison using smooth plates versus serrated plates is shown in Figure S3 for two samples: the DN (only water) and the corresponding sample containing Ecosurf. For the Ecosurf sample, the change in plates had a remarkable effect on both frequency and stress sweeps (Figure S3b, d). One can also note the large error bars in the frequency sweep for this sample tested with smooth plates, while the error bars for the tests with serrated plates are very small (smaller than the symbol). Figure S3a, c also illustrate that the sample DN with only water in both tests (smooth and serrated plates) has very small error bars, and the change in plates has no effect on the results. Both stiffness levels were well within 10% of the average value, indicating that there is no need for correction factors when working with the serrated plates. Furthermore, with the serrated plates, all the gels displayed a very similar viscoelastic range, reaching almost 100% strain. All further tests discussed in this study were recorded using the serrated plates

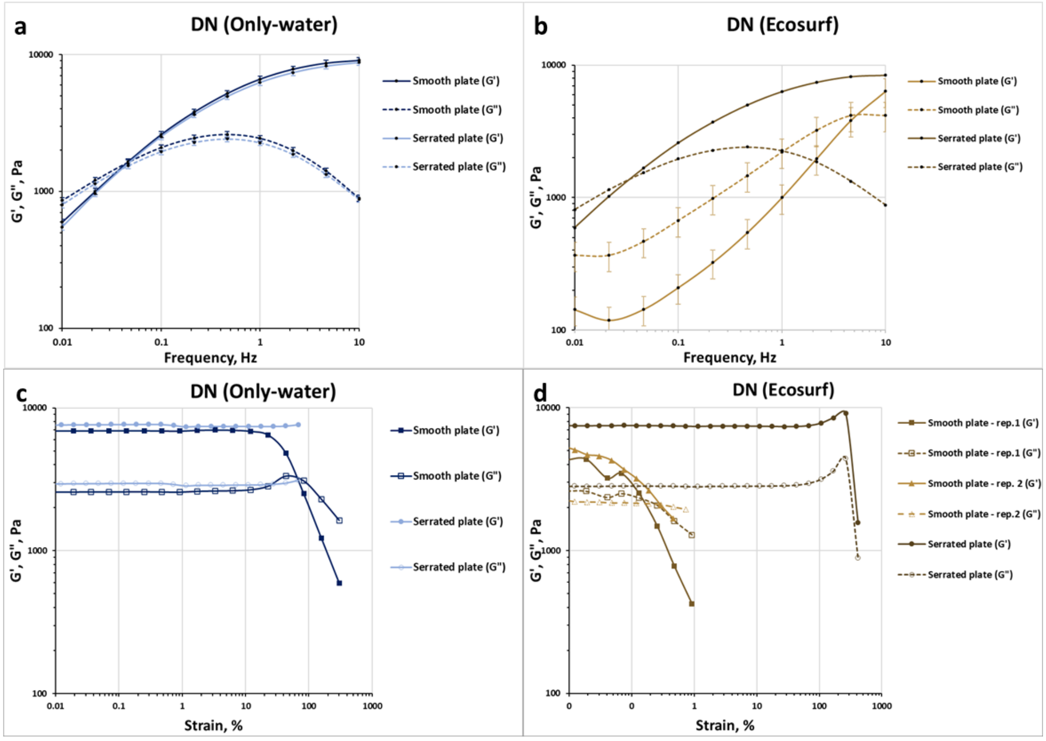

Figure S3: (a) and (b) Comparison of smooth and serrated plates in frequency for two hydrogel formulations. (c) and (d) Comparison of stress sweeps for the same hydrogel formulations.

Supplementary data on micro-FTIR

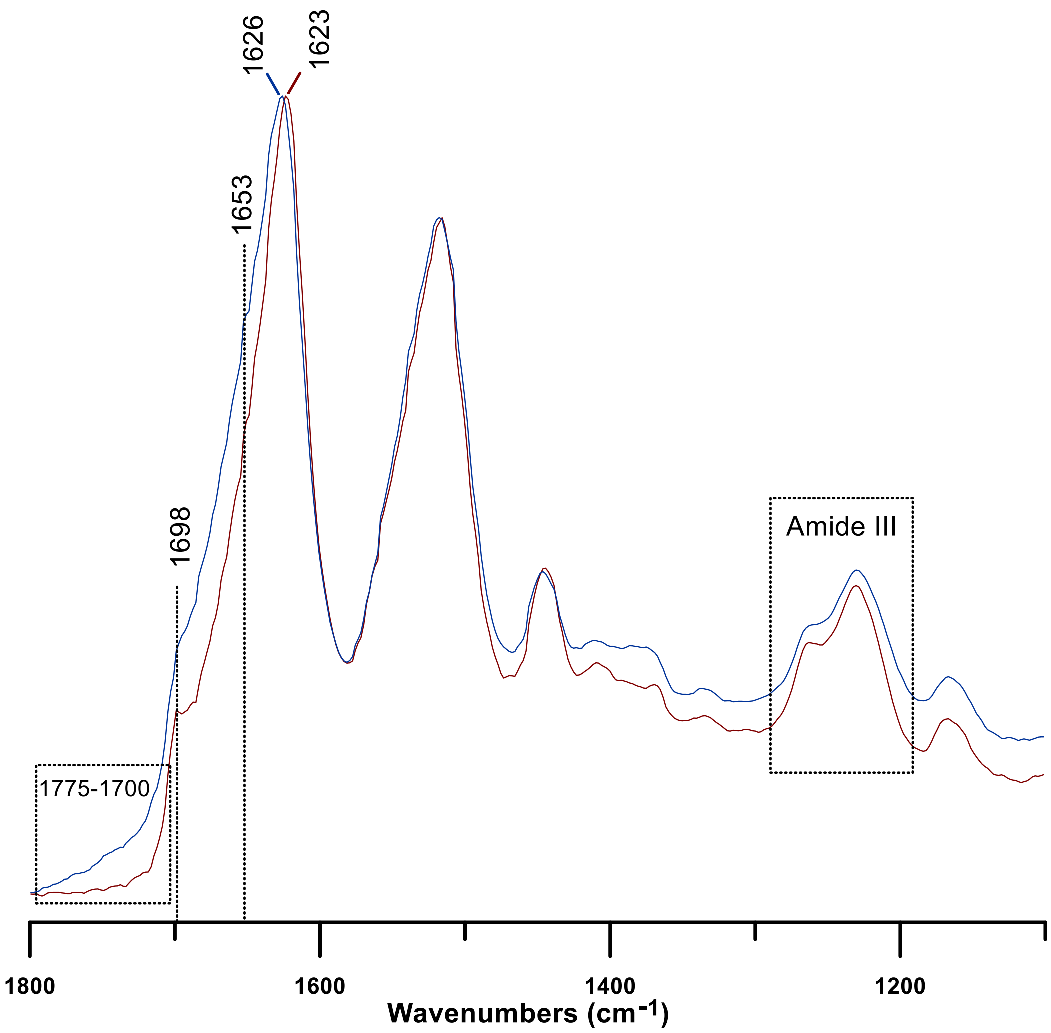

Figure S4: ATR-FTIR spectra of artificially aged (blue) and unaged silk (red) in the range of 1800-1100 cm^{-1}.

It was decided to investigate the presence of PVA-B gel residues on the treated SMS. For this reason, SMS treated with PVA-B loaded with only water (tamping approach) was measured by micro-FTIR. The collected spectra do not show the main characteristic bands of the gel, i.e., the peaks at 2910, 2861, and 1107 cm^{-1} (see Figure S5). Nevertheless, the SMS treated with gels loaded with Ecosurf was investigated for the detection of residues of the surfactant. The Ecosurf spectra exhibited a sharp and strong peak at 1098 cm^{-1}, which could be attributed to the C-O-C asymmetric stretch of the ethoxylates. This definitive peak is totally absent in the spectra collected from an SMS treated with DN ‘Ecosurf’ for 5 min contact time (see Figure S6).

https://doi.org/10.3390/gels12010097

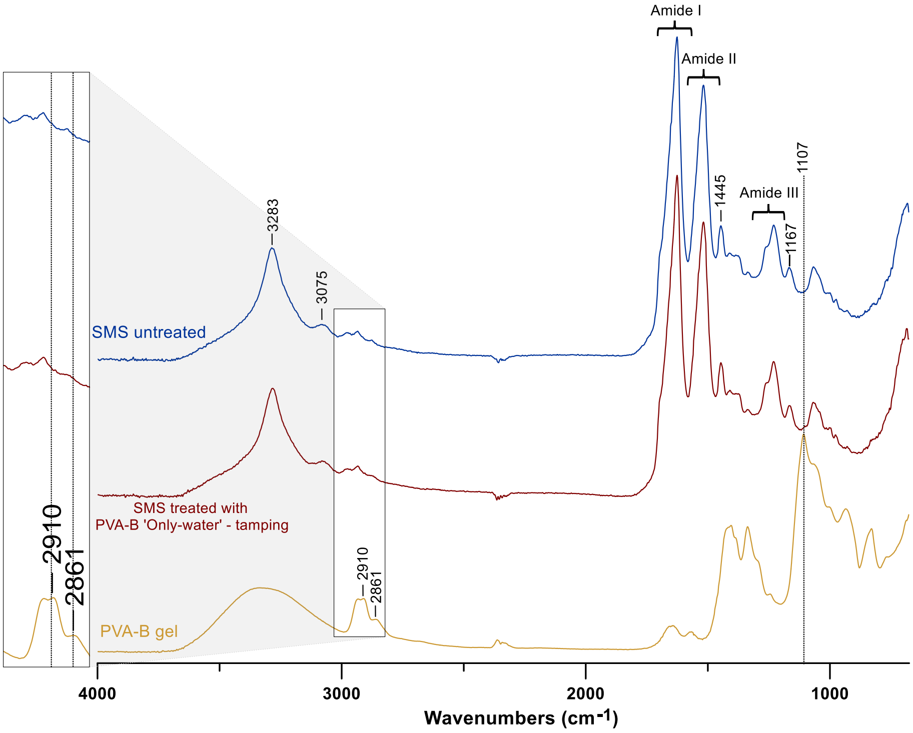

Figure S5: ATR-FTIR spectra of untreated SMS, SMS tamped with PVA-B loaded with only water, and PVA-B gel. A detail of the spectra in the 2800-3000 cm^{-1} range is shown on the left. It indicates that the characteristic peaks at 2910 and 2861 cm^{-1} of the PVA-B gel are absent in the spectrum of the treated SMS, as is the intense peak at 1107 cm^{-1} indicated by the dashed line.

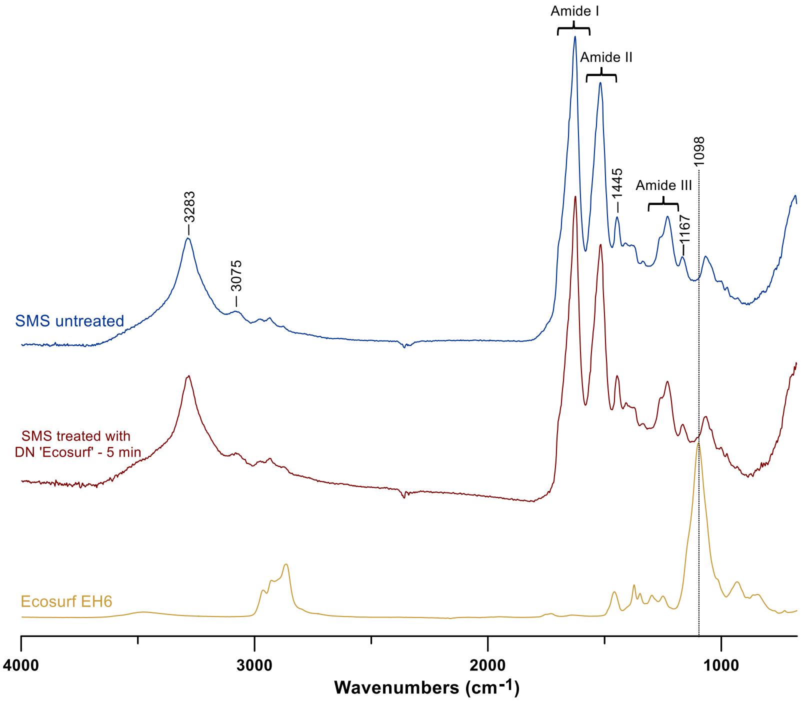

Figure S6: ATR-FTIR spectra of untreated SMS, SMS treated with DN with Ecosurf, and Ecosurf EH-6. The treated SMS does not show the definitive peak of the Ecosurf at 1098 cm^{-1}.

Disclaimer/Publisher's Note: The statements, opinions and data contained in all publications are solely those of the individual author(s) and contributor(s) and not of MDPI and/or the editor(s). MDPI and/or the editor(s) disclaim responsibility for any injury to people or property resulting from any ideas, methods, instructions or products referred to in the content.

https://doi.org/10.3390/gels12010097
